# Examining the application of innovative ELT strategies in primary school EFL classrooms

**DOI:** 10.1371/journal.pone.0328902

**Published:** 2025-08-25

**Authors:** Belyihun Muchie, Abiy Yigzaw, Birhanu Simegn

**Affiliations:** Department of Language Education, School of Teacher Education, College of Education, Bahir Dar University, Bahir Dar, Ethiopia; Faculty of Electrical Engineering, Computer Science and Information Technology Osijek, CROATIA

## Abstract

English language teaching strategies determine the practicality of effective language instruction. Their effectiveness, however, was not directly examined in the context of Ethiopian primary schools. This study aimed to explore primary school EFL teachers’ innovative teaching strategies employment. The study employed a descriptive survey research design with a convergent mixed methods approach. Ten primary schools were selected conveniently, including 60 teachers. They were selected based on the accessibility of the schools and the inclusion of all English teachers. Classroom observation, questionnaire and document analysis were used to collect data. The quantitative data were analyzed in percentages, whereas the qualitative data were analyzed using thematic analysis techniques. The finding revealed that primary school English language teachers were not teaching English using innovative teaching strategies: cooperative learning, task-based learning, project-based learning, differentiated learning, problem-solving, critical thinking, and technology integration. Hence, the alignment of teaching strategies with the syllabus of English was mismatched. Although there were challenges in employing innovative teaching strategies, teachers’ commitment was found to be less. Therefore, it was concluded that primary school English language teachers were not employing innovative ELT strategies in Ethiopian primary schools.

## 1. Introduction

English as a Foreign Language (EFL) instruction is evolving rapidly, with a growing focus on pedagogical innovation to enhance teaching effectiveness and student engagement. Since the demand for English proficiency increases globally, educators are encouraged to move beyond traditional methods and adopt learner-centered strategies [1]. This shift is particularly fundamental in Ethiopian primary schools, where English serves both as a subject and a medium of instruction (in some regions). The success of EFL teaching depends on how well teachers integrate innovative teaching approaches that cater to students’ linguistic and cognitive needs [2].

Therefore, EFL teachers are responsible to employ engaging English Language Teaching (ELT) strategies that foster interactive lessons, and promote active learning. As [3] emphasizes, effective English teaching is rooted in strategic choices and their implementation in appropriate settings. Teaching strategies serve as the realization procedures of broader teaching methods, and approaches that they provide flexibility for teachers to fit their instruction based on students’ needs and classroom activities [4,5].

EFL teachers can integrate various language teaching strategies by adapting and interchanging them based on contextual factors. They strategically combine multiple teaching strategies within a structured framework that fosters creativity and innovation in the classroom, reinforcing students’ learning experiences. Given the essential role of ELT strategies, their effective use becomes a necessary component of English language instruction. This study explored how Ethiopian primary school EFL teachers perceive and implement innovative ELT strategies in their classrooms. Examining the extent to which modern methodologies are integrated into teaching practices, the research highlights both challenges and opportunities in adopting new approaches. The findings contribute to ongoing discussions on EFL instruction, and provide insights for policy recommendations regarding teacher training and curriculum development.

## 2. Statement of the problem

Extensive discourse has marked the history of language teaching. The argument has centred on effectively teaching the English language within the context of learners’ diverse learning needs. The shift from the era of methodological approaches to the post-method era indicates that the practice of English language teaching has not yet achieved a satisfactory level of practicality [6]. Then, [4] proposed a further shift, moving away from the emphasis on methods and toward a focus on the strategic use of language teaching practice. According to his assumption, teaching methods are inherently dependent on the employment of effective strategies in the classroom. Concentrating on ELT strategies, the practice of language instruction can be made more practical and adaptable across various contexts. Thus, the role of an EFL teacher should be refashioned and teaching strategies would be the concern of this role change [7].

In Ethiopian primary schools, English as a Foreign Language (EFL) is taught as a subject from Grades 1–6 and serves as a medium of instruction in some regions. The syllabus and textbook are designed to promote communicative language teaching, incorporating interactive learning tasks to enhance students’ language skills. However, despite certain limitations in the teaching materials, the most pressing challenge in English instruction lies in the actual teaching practices of EFL teachers. The quality of classroom instruction and the extent to which teachers effectively implement contemporary methodologies require thorough investigation to ensure their professional competencies remain up to date.

At the primary education level, children possess a natural ability to acquire languages more easily [8]. Consequently, substantial efforts must be directed toward enhancing the effectiveness of English instruction at this stage. In Ethiopia, concerns about the quality of English teaching have intensified, particularly due to the declining number of Grade 12 students successfully enrolling in universities. A significant number of students complete elementary school without attaining fundamental literacy and numeracy skills [9]. According to [10], the challenges faced in secondary education can be traced back to early childhood learning experiences. This gap in foundational education has long-term implications for their academic progress and overall educational outcomes.

Besides, English has been identified as a significant barrier contributing to students’ poor academic performance, because it serves as the medium of instruction in higher grades. Therefore, primary school EFL teachers must demonstrate a high level of proficiency in both pedagogical content knowledge and classroom practice. Their instructional approaches play a crucial role in addressing the challenges of teaching English, both as a subject and as a medium of instruction. Enhancing their teaching methodologies is imperative to improving students’ language competencies and overall academic success.

Despite extensive research on effective EFL pedagogy, little is known about how Ethiopian primary school teachers implement and adapt innovative teaching strategies in their unique contexts. While methods like communicative language teaching, task-based language teaching, and technology-enhanced learning are widely promoted, their practical application in Ethiopian classrooms remains unclear. More recent research works have been conducted to refine the philosophy of English teaching.

To compare the global ELT practices of 2010 and 2020, [11] conducted a study and they claimed that teachers use teacher-led teaching approaches in the global contexts. [12] also collected data from journals to explain innovative teaching strategies in teaching English as a foreign language. This systematic review identified three innovative teaching strategies: cooperative learning strategy, problem-based learning strategy and project-based learning strategy. It highlights effective teaching of English should integrate technology, multimedia resources, and elements of culture with innovative teaching strategies. Moreover, [13] explored innovative and creative English teaching strategies. The study outlined the benefits and challenges of various strategies by reviewing previous studies systematically. Despite the challenges, the findings demonstrated how instructional flexibility, student-centred learning, technological integration, and careful lesson design all work together to improve students’ critical thinking, problem-solving abilities, and general motivation in English language classes.

Online teaching and learning have become increasingly prevalent in recent years, prompting shifts in pedagogical approaches. For instance, [14] investigated an online pedagogical approach for teaching grammar at the primary level. It was revealed that while online strategies enable the integration of diverse instructional methods, teachers encountered difficulties in assessing students’ comprehension and learning of grammar content. Additionally, research has explored strategies to support the transition back to face-to-face instruction. In the Philippines, [15] conducted a study titled *Innovative Strategies for Teaching English* to address learning gaps and enhance instructional effectiveness in traditional classrooms. Findings suggested that learning activities should incorporate students’ daily experiences, teachers should employ varied instructional strategies, and scaffolding techniques should be utilized to support learners effectively.

The above studies primarily focused on specific skills teaching strategies. [16] as an example, studied language teaching strategies’ impact on third-grade students’ reading outcomes and reading interest. Its finding revealed that there is a correlation among students’ reading interest, vocabulary knowledge and text comprehension. [14] also studied the pedagogical approach to the teaching of grammar skills. These studies did not comprehensively explore the actual teaching strategies employed in EFL classrooms. Besides, the findings of some studies suggest limitations in their ability to directly observe teachers’ instructional strategies in the classroom [17]; others used questionnaires [15], and interviews [13] as data collection instruments to explore classroom-teaching strategies. It is important to note that the methodologies employed in these studies were restricted to one or two data collection instruments. It potentially limits the richness of the data and the depth of understanding achieved through a mixed-methods approach.

The current study was, therefore, different in terms of purpose, methodology and geographical contexts. It aimed to explore primary school EFL teachers’ teaching strategies in their classrooms. It delimited on exploring the applications of innovative English language teaching strategies to teach English. It was also different in terms of data collection instruments, using classroom observation, questionnaires and document analysis. The participant teachers were selected from 10 primary schools at Woreta city administration. Geographically, the context of this study was at Ethiopian primary schools, in which Amharic is a native language, and English is a foreign language for both teachers and students. For the best choice and usage of ELT strategies in the context of learners, a continual investigation is invaluable.

Further, drawing upon a preliminary study that identified potential limitations in the use of diverse teaching strategies, this research investigated the instructional practices employed by primary school English language teachers at Woreta city administration. The preliminary findings suggested a need for a more in-depth exploration, as limited strategic application could hinder the effectiveness of English language instruction in this context. The current study aimed to explore primary school English language teachers’ teaching strategy utilization in EFL classrooms at Woreta city administration primary schools. Specifically, it intended to:

a determine the creative teaching methods in EFL classes by English language instructors in primary schools.
b examine how well they fit the tenets and methods of modern language instruction.
c asses how primary English language teachers view the advantages and difficulties of putting creative teaching methods into practice.

## 3. Literature review

### 3.1. ELT strategies

Innovative in this study, refers to strategies and techniques that improve the learning process by introducing novel concepts, procedures, or technological advancements. It implies that the most important elements to improve English learning are creativity, flexibility, technological integration, and collaborative learning. They allow students to use their language abilities in real-world situations. These strategies demonstrate different contemporary teaching methodologies. There are many creative ELT strategies in general though we highlight the most popular approaches in EFL classrooms.

EFL teachers teach language skills using different strategies within different contexts. They have the opportunity to implement a variety of teaching strategies. Role-plays bring the real world into the classroom context, which develops students’ language fluency and creates collaborative learning experiences [17]. Teaching through storytelling appeals to learners to listen to stories, and it enables them to promote their listening, communication, and vocabulary skills [18]. Teachers can also teach using audiovisual aids in the classroom. Radios and tape recorders are used to practice listening, speaking, and pronunciation skills, and visual aids like charts, maps, filmstrips, pictures, flashcards, graphs, diagrams, posters, etc. assist students in catching their attention in long-term memory.

Teachers may teach through the JAM (Just a Minute) teaching strategy to engage students in speaking and vocabulary activities [19]. The teacher allows students to speak for one minute without stopping using a certain topic, but the speaker is restricted not to repeating and deviating from the opponents’ ideas. The common teaching strategy even in other subjects is group discussion. In teaching English, it is helpful to make cooperative learning, to exchange ideas one to the other. In this case, students will improve their critical thinking, analytical thinking, listening and speaking skills [20]. Games are also fantastic ways of teaching English to learn vocabulary pronounce words, and develop sentences and grammar [21]. Flashcards make games more interesting to teach language skills. Teachers’ role is to identify the appropriate games matching with language activities and the age of the students.

### 3.2. Opportunities and challenges of innovative ELT strategies

Teachers adapt and interchange preferable strategies by planning teaching objectives. The good quality of ELT strategies is not rigid to a formal regulation. These strategies explain broad pedagogical techniques that can be integrated into the specificities of teaching events and environments. Consequently, the teacher’s pedagogical approach, creativity, and individual characteristics become prominently reflected in the chosen strategies. They have functions to structure the teaching contexts in the classroom. The teacher make a smooth connection between the students and learning activities by using modelling teaching strategies, which initiates students’ motivation for the tasks.

In maximizing these strategies’ effectiveness, teachers need to consider the diverse learning styles and contextual factors that influence students’ engagement and comprehension. Supporting this, [22] recommends that EFL teachers should adapt strategies based on their learners’ context, considering various factors in choosing effective strategies. Every student has a unique learning style in a classroom, and the teacher will try to accommodate these differences by employing a range of strategies. Students from diverse cultural backgrounds may also bring to the classroom prior language skills, learning preferences, and cultural norms [23]. Individual differences in language acquisition persist even among students within the same grade level and cultural background. To address this heterogeneity, a multifaceted approach to teaching strategies is optimal integrating diverse ELT strategies into learning tasks. Teachers can cater to the varied learning needs and acquisition styles of students, fostering inclusive language acquisition. When planning lessons, teachers take these things into emphasis by adapting their teaching methods and incorporating culturally relevant resources to create a more inclusive learning environment for students from diverse backgrounds.

Besides students’ demographics, several other factors affect the particular teaching strategies used. Large class sizes may make it more difficult to engage students in interactive activities, which may force teachers to adopt teacher-centred strategies like lectures or presentations [24]. On the other hand, even in larger classes, having access to interactive whiteboards or tablets can lead to engaging activities though the availability of resources is a limitation [25]. Curriculum requirements may also be relevant in that certain curricula allow for greater flexibility, but others may be more restrictive in what the teacher can strategically choose from. In the end, teachers’ exposure to various approaches and training also influence their strategy choices. [26] reflects that teachers who have employed different approaches will exhibit greater flexibility and adeptness in selecting the most efficient techniques for their specific context.

In conclusion, Innovative English Language Teaching (ELT) strategies encompass a range of student-centred approaches that integrate creativity, flexibility, technological advancements, and collaborative learning to enhance language acquisition. These strategies include role-plays, storytelling, audiovisual aids, Just a Minute (JAM) activities, group discussions, and games, all of which facilitate authentic language use and engagement [18,19,21]. Teachers play a pivotal role in adapting these methodologies based on classroom contexts and student needs, emphasizing the importance of pedagogical creativity and contextual flexibility. However, the effectiveness of these strategies is influenced by factors such as students’ learning styles, cultural backgrounds, classroom size, technological resources, and curriculum constraints [22,23]. To address these challenges, teachers must employ a multifaceted approach, integrating diverse ELT strategies and culturally relevant materials to foster inclusive learning environments [22]. Furthermore, professional development and exposure to varied teaching methodologies enhance teachers’ adaptability and efficacy in implementing innovative ELT strategies [26]. Ultimately, the success of these approaches depends on teachers’ ability to balance pedagogical techniques with contextual constraints to optimize language learning outcomes.

## 4. Methodology

### 4.1. Design of the study

This study adopted a descriptive research design within a convergent mixed-methods approach leveraging both data types to present a holistic view of ELT strategies in EFL classrooms. This approach integrates quantitative and qualitative data to provide a comprehensive understanding of teaching strategies [27]. Given the complexity of language instruction, it enables cross-validation of findings and a deeper exploration of pedagogical practices.

### 4.2. Participants of the study

The participants of this study were primary school English language teachers teaching in the Woreta City Administration. There are 10 primary schools in the city. A total of 60 English language teachers, who were teaching in grades 1 to 6 were selected as the study sample. A convenience sampling technique was employed due to the accessibility and proximity of the participants to the researcher. While the number of English teachers varied across schools, all English teachers within the specified grade levels were included, as the total number was manageable for the study. It was ensured that the perspectives of all available English teachers in the area were represented, thereby enhancing the comprehensiveness of the data.

Ethical considerations of this study were issued by Bahir Dar University, College of Education Institutional Research Review Committee (IRRC). The committee decided on 1/11/2024 and gave a meeting number 7/24, protocol number 001957, and assigned number 014. The researchers employed a combination of classroom observations, questionnaires, and document analysis as data collection tools. These instruments were validated and assessed by a review committee at Bahir Dar University. The Institutional Research Review Committee (IRRC) approved data collection on November 1, 2024.

Data collection occurred from November 7 to December 11, 2024. In the context of research conducted at our university, additional participant consent forms are not required, as the research is designed to pose no harm to participants. To affirm the ethical nature of this study, the IRRC at Bahir Dar University, College of Education, has approved the study’s objectives and assessed the potential risks to participants. The committee ensured that all necessary protocols for data collection were followed in the primary schools under the Woreta City Administration.

### 4.3. Data gathering instruments

Data were collected through classroom observations, questionnaires and document analysis. Observations provided firsthand insights into instructional practices, while questionnaires captured teachers’ perspectives. Document analysis supplemented these findings by revealing instructional planning and implementation. Triangulating these data sources ensured a robust examination of ELT strategies.

Classroom observation was held to observe the classroom applications of ELT strategies. It was employed to identify macro and/or micro-teaching strategies, which primary English language teachers utilized in their classroom setting. The observers were non-participant observers (complete observers). Semi-structured observation was employed, because the heterogeneous nature of the teaching process involves various implications to be included for construct validity. The observation checklist was a ‘yes/no’ type, at the same time the field notes were recorded alongside the checklist. The observation checklist was adapted from other models very related to EFL teaching strategies. For data adequacy, the observers used stimulated recall through audio, video, and audio-visual recordings of classroom teachers while they were teaching. To ensure accuracy and minimize interpretation bias, the data were transcribed based on an observation checklist and field notes. Additionally, multiple raters were involved in the transcription and analysis process to enhance reliability. Triangulation with teachers’ reflections and peer debriefing further helped control potential biases in interpreting the recordings.

The questionnaire aimed to identify innovative teaching strategies employed by primary English language teachers. It also categorized the alignment of various language-teaching strategies with contemporary pedagogical approaches and principles in language education. Moreover, the questionnaire realized the challenges and benefits of implementing innovative teaching strategies among primary English language teachers. It consists of qualitative data with open-ended types.

The researchers also analyzed teaching documents as a source of secondary data. These documents helped them to substantiate the observation and questionnaire verifying the findings of the study [28,29]. Analyzing teachers’ teaching documents primarily focused on identifying actual teaching strategies and aligning them to the teaching approaches and theories. These documents were categorized into lesson plans, teaching materials, teachers’ reflective journals, and professional development records. The researchers obtained reliable data to analyze ELT strategies from these pre-existing documents to finish their study [28]. The documents made a crosschecking of teaching strategies at their plan level with their classroom implementation level.

### 4.4. Data collecting procedures

The nature of the study permitted to keep the data complications of the three collecting tools. Observing English teachers, at the first stage was a realistic approach that the participants’ biases were decreased. It was the initiative process of the next steps. The researchers got advantages and inputs for good qualities of the questionnaire and document analysis. The observers observed all participant teachers by scheduling two teachers per day. Next, participants responded to the questionnaires. The researcher chose 20 participants from 60 teachers, in a simple systematic randomization for the document analysis work. Then, their documents were organized and analyzed to look at the data triangulation between observation and questionnaire data sets.

## 5. Data analysis methods

The researchers analyzed the data with quantitative and qualitative techniques. Quantitative data were analyzed using percentages to represent the distribution applications of teaching strategies. The percentages data found in the observation, showed the utilization of innovative teaching strategies in EFL classrooms. The reliability of the observation was checked through inter-rater reliability. The consistency of observation data made by the researcher and an expert were compared, and their scores were compared using Cohen’s Kappa coefficient (0.80) to ensure the agreement between their ratings.

For qualitative analysis, we used thematic Analysis. This method allowed us to identify key patterns and themes in the participants’ descriptions of innovative teaching strategies. The process involved familiarization with the data, coding, generating themes, reviewing and defining them, and finally reporting the results in relation to the research questions. Thematic Analysis was chosen for its flexibility and suitability in exploring the lived teaching practices and pedagogical innovations employed by primary school English teachers.

To ensure reliability in qualitative analysis, an inter-coder reliability check was conducted. The researchers independently coded the data and compared their results to maintain consistency, and Cohen’s Kappa was found to be 0.78, which is a substantial agreement [29]. To enhance the validity of the findings, data triangulation was employed by integrating multiple data sources, such as observations, questionnaires, and document analysis, ensuring cross-verification of information, reducing bias, and increasing the credibility of the results.

## 6. Results and analysis

### 6.1. Application of teaching strategies

A classroom observation was held using semi-structured checklist items to realize primary school EFL teachers’ classroom teaching strategies. The data were analyzed in three themes such as lesson structure observation, instructional strategies, and instructional tools and resources. The following three graphs show the percentage analysis of innovative ELT strategies application in EFL classroom.

**Fig 1:**
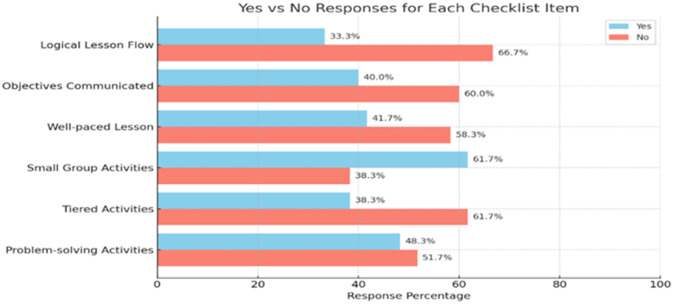
Lesson structure observation.

Fig 1 illustrates the percentage of various teaching strategies employment. The observations data showed that 66.7% of lessons lacked logical flow, 58.3% were poorly paced. 60% of the lesson objectives did not communicate to students. The lesson content often failed to connect with students’ learning backgrounds. Furthermore, 51.7% of activities were not problem-solving tasks, and 61.7% lacked complexity nature.

**Fig 2:**
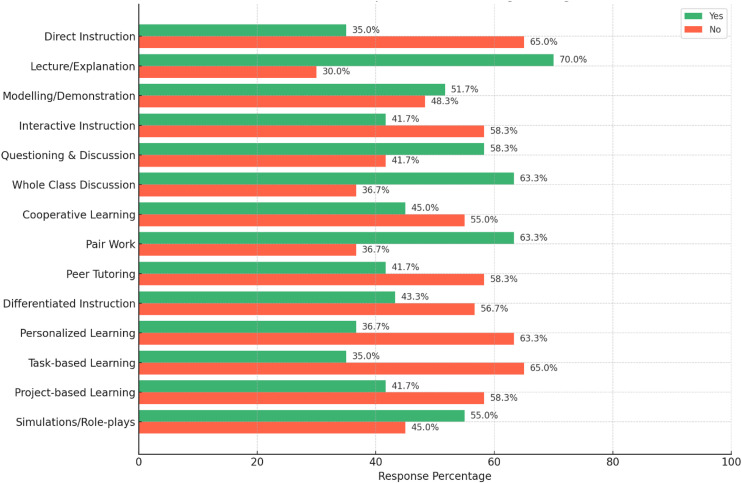
Instructional strategies.

Fig 2 shows the instructional strategies utilized by primary school English teachers in their classrooms. It revealed, most teachers frequently used lecturing (70%) and question-and-discussion methods (58.3%). However, 65% did not apply direct teaching instructions to develop language skills or introduce new knowledge. Direct instruction implicates structured, explicit guidance fitted to student needs [30]. In their teaching, whole-class discussions were common, but they lacked interactive demonstration of tasks (58.3%). Thus, it led to weak classroom collaboration (55.0%).

Primary school English teachers frequently apply pair work (63.3%) and small group activities (61.7%). They used them for grammar instruction, mainly to teach language structures. Nonetheless, peer tutoring was not used collaboratively (58.3%). Differentiated instruction was largely absent (56.7%). It neglected students’ needs and interests (63.3%). Task-based (65.0%) and project-based learning (58.3%) were also not practised in their classrooms. Role-plays (55.0%) were used though they relied heavily on scripted conversations.

**Fig 3:**
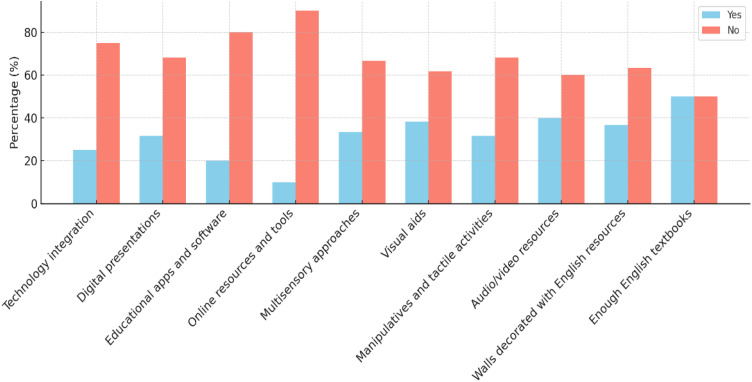
Instructional tools and resources.

Fig 3 reveals a major gap in the integration of technology into teaching strategies. As it has been indicted in the graph, 75% of instruction lacked technological support. 68.3% of teachers did not use digital presentations and 80% did not use educational apps or software. The observation data showed that 90% of teachers did not use online tools to support language practice, pointing to limited adoption of technology-enhanced methods in EFL instruction. Additionally, many teachers were not using multisensory strategies. Visual aids (61.7%) and audio/video resources (60%) were rarely employed, leading to 66.7% of teachers failing to engage students through multiple senses. Consequently, student participation was low, as tasks were not hands-on or interactive (68.3%). Teachers also faced challenges in assessing student understanding, likely due to weak instructional delivery.

### 6.2. Questionnaire data analysis

#### 6.2.1 Teaching strategies utilization.

A questionnaire was administered to teachers to identify the types of teaching strategies they commonly employed in English instruction. The responses revealed that 53 teachers frequently used lecturing, 39 employed question-and-answer techniques, 26 implemented group discussions, and 13 utilized role-play strategies. Despite the frequent use of these methods, many teachers expressed dissatisfaction with their effectiveness in enhancing student learning. When asked to rate the effectiveness of these strategies on a scale from 1 (not effective) to 5 (highly effective), 42 teachers rated them as 1, 11 as 2, and 7 as 3.

In contrast, teachers showed strong interest in using alternative teaching strategies such as cooperative learning, task-based learning, project-based learning, differentiated instruction, problem-solving, critical thinking, and technology integration. They believed these approaches could enhance student engagement, were easy to prepare and implement, supported speaking practice, and aligned well with students’ proficiency levels. Teacher 17 for example, responded:

Yes of course, I am interested to apply cooperative learning strategies in my classroom. Because they actively engage students and help them to build communication skills. In the past, I have not used them regularly. However, I believe they will improve engagement and speaking practice in my lesson.

#### 6.2.2. Relevance of teaching strategies to contemporary approaches.

Their responses regarding balancing their personal teaching preferences with the requirements of the curriculum, were not satisfactory. The teachers neither fully implemented nor do they adapted the existing strategies appropriately. As confirmed by 56 of the teachers, they were often teaching selecting content based on personal preference rather than pedagogical relevance. This practice resulted in inconsistent and unstructured teaching. They reflected that they were not consistently updating or applying innovative teaching strategies. The strategies employed did not align with the curriculum, as the teachers failed to incorporate the innovative approaches recommended in the syllabus. The most participants rated to very low and low alignments to the curriculum. A teacher (T-28) expressed this gap, as:

My teaching strategy preferences are not in line with the syllabus’ designation because tasks in the syllabus are not interesting to teach my students. The tasks are not about language structures; rather they are more focused on strategies and techniques of practicing skills. My students are not interested to learn the theories of learning English; they are willing for the grammar contents of the subject.

#### 6.2.3. Teachers’ perceptions of benefits and challenges of innovative teaching strategies.

The teachers also admitted the benefits of using innovative teaching strategies. Precisely, 52 teachers conveyed that such strategies improve students’ linguistic competence and critical thinking skills. According to 47 teachers, these methods also increase student engagement and inspiration. Additionally, 34 teachers noted that innovative strategies improve students’ speaking and communication capabilities, while 39 indicated that they enhance classroom interaction. Furthermore, 54 teachers perceived that these strategies contribute to the development of a more student-centered learning situation.

However, they stated the major challenges to employ these strategies in their EFL classroom. The teachers were struggling with overcrowded classrooms and rigid schedules to employ innovative teaching strategies. Instructional constraints were also evident, as teachers demonstrated varying degrees of limited theoretical knowledge regarding innovative teaching strategies. Some were completely unfamiliar with methods like differentiated instruction and task-based learning. Others had only a surface-level understanding. Even with some awareness of cooperative learning and problem-solving methods, teachers often found it challenging to design appropriate classroom activities to practice the language skills.

Teachers also faced severe shortages of essential resources needed to implement innovative teaching strategies. Schools had no access to digital tools such as computers, projectors, or internet services. This made it difficult to use technology-based methods like digital presentations and audio-visual aids. Moreover, limited professional development materials and training opportunities left teachers without proper guidance on approaches like task-based, problem-solving, and cooperative learning. The constraints significantly hindered innovative teaching and negatively affected student engagement and language learning outcomes.

### 6.3. Documents analysis

Lesson planning serves as the foundation for all teaching activities, aligning learning tasks with appropriate strategies based on students’ levels [13]. The researchers analyzed teachers’ lesson plans to examine the strategies employed in the classrooms. In the plans the objectives were found to be less engaging and inadequately aligned with students’ motivational needs. They reflected a teacher-centred view of English as knowledge delivered rather than co-constructed. Student engagement and participation were limited. [31] argues that teaching should prioritize learners’ needs over rigid adherence to a lesson plan.

Teachers have recorded self-evaluations of their daily reflective journals. The journals showed a repetitive use of similar approaches, reflecting a lack of motivation to teach English. Teachers viewed English instruction as serving only short-term purposes and showed little concern for adapting strategies to different lessons. This was largely due to limited awareness of effective teaching methods and strategies for EFL. The journals also highlighted challenges such as students’ weak learning backgrounds and large class sizes, which hindered the implementation of innovative teaching strategies.

## 7. Discussion

In ELT, the most common innovative teaching strategies include cooperative learning, task-based learning, project-based learning, differentiated instruction, problem-solving, critical thinking and technology integration [4]. This study aimed to identify innovative teaching strategies employed by primary EFL teachers, analyze their alignment with contemporary language teaching principles, and evaluate teachers’ perceptions of the challenges and benefits associated with implementing these strategies. Primary school EFL teachers face challenges not only in utilizing available materials but also in aligning their pedagogical strategies with students’ learning preferences. Teacher-centred approaches continue to dominate, emphasizing structural explanations. This rigid instructional focus on grammar instruction and rote memorization limits the implementation of diversified student-centred teaching strategies. Ultimately, this hinders effective and engaging language learning experiences.

It was found that there was prevalence of teacher-led instruction and a strong focus on structural explanations, which suggested a limited adaptation of innovative teaching strategies. This lack of variation in instructional strategies is further reflected in classroom observations, where teachers predominantly relied on lecturing. While this finding aligns with some previous research [32,33], it contrasts with studies advocating for more student-centered methodologies [10,13,34–36].

In the context of Sub-Saharan Africa, the implementation of such strategies is hindered by several critical challenges, including restricted access to high-quality instructional resources, overcrowded classrooms, diverse linguistic backgrounds, and inadequate teacher training [37]. A similar situation is evident in Ethiopian English teaching, where there is a high demand for English language proficiency, yet student achievement remains low [38–40]. One of the primary reasons for this low achievement is the continued reliance on teacher-centred instructional approaches. Despite the ongoing push for communicative language teaching, these findings highlight the gap between recommended practices and actual classroom implementation. It emphasizes the need for greater adoption of interactive teaching strategies.

The dominance of teacher-centred instruction limits student engagement and active participation. It restricts student interaction, reduce opportunities for meaningful practice that impedes language skill development. Integrating interactive and innovative methodologies-such as technology-enhanced learning, student-centred strategies, and culturally responsive teaching can foster a more engaging and effective EFL learning. However, the successful adoption of these approaches relies on comprehensive policy support, continuous teacher training, and the availability of necessary socio-economic and infrastructural resources.

Teachers are expected to serve as proficient language models by demonstrating tasks. Nonetheless, the effective implementation of modelling and demonstration-based teaching strategies remains a challenge for the teachers. Moreover, interactive instructional strategies, such as teacher-student and student-student interactions, were notably underutilized. While teachers incorporated small group discussions, role-plays, and pair work in certain lessons, these methods were not fully leveraged to foster meaningful engagement. The primary constraint, as perceived by teachers, was their lack of experience in effectively employing these strategies, resulting in minimal facilitation of active student participation. Teachers failed to differentiate instructional tasks according to individual learning needs. Instead, one-size-fits-all approach was employed, disregarding the complexities of language acquisition. Although textbook tasks were designed to engage students at varying proficiency levels, their implementation was ineffective due to teachers’ limited expertise in task-based teaching methodologies.

A critical deficiency in English instruction was the ineffective use of teaching resources. This study highlighted multiple challenges, including the tension between strict curriculum adherence and the integration of supplementary materials. Research [41] indicates that both teachers and students who sought to incorporate additional resources risked deviating from the prescribed curriculum. Rigid compliance often restricted the effective use of diverse teaching materials. This ongoing dilemma has hindered the advancement of English language instruction in Ethiopian primary schools. It has limited educators’ ability to adopt innovative pedagogical strategies.

In enhancing students’ engagement and language proficiency, the integration of technology into English language instruction is increasingly recognized as a crucial factor [42]. Therefore, fostering innovation and creativity, technology enriches teaching strategies and broadens the scope for effective learning. However, teachers’ views of technology use often persist rooted in traditional pedagogical approaches, influenced by their general awareness with digital resources. Teachers tend to overestimate the challenges associated with technology integration. Particularly, they feel frustrated regarding resource availability and procedural complexities. This study revealed that the actual application of technology in ELT fell short of expectations. Teachers primarily relied on conventional instructional materials, such as graph paper and fabric, for creating charts and diagrams. They use these materials mainly for lessons on sentence structure and grammar patterns.

There has been a disconnected persists between the theoretical underpinnings of the curriculum and its application in classrooms. Teachers do not fully utilize the prescribed framework, resulting in inconsistent instructional practices. The curriculum emphasizes competency-based learning through structured procedures, interactive and differentiated instruction, and scaffolded skill development, aiming to enhance students’ oral proficiency and overall language acquisition. The curriculum focuses on real-life language use and innovative English Language Teaching (ELT) strategies. However, primary school English teachers predominantly rely on lecturing, questioning, group discussions, and role-playing. Their reluctance to adopt more flexible and interactive teaching strategies limits student engagement and motivation. This disparity suggests a misalignment between curriculum recommendations and classroom practices. It is often attributed to teachers’ skill deficiencies and the challenging educational environment.

The study highlights that primary school English teachers faced significant challenges in implementing innovative ELT strategies due to students’ habituation to lecture-based instruction. This passive learning situation has impeded the adoption of ELT strategies. The findings indicated that fostering these strategies requires extensive teacher training and the integration of appropriate teaching resources to align instructional strategies with curriculum objectives.

Moreover, this study has identified a misalignment between teachers’ instructional practices and contemporary pedagogical frameworks, necessitating deliberate efforts to incorporate innovative teaching strategies. Several barriers hindered this transition. Firstly, while teachers exhibited theoretical awareness of innovative methodologies, their pedagogical knowledge remained insufficient for effective implementation. Secondly, student expectations and attitudes reinforced passive learning tendencies, further limiting the adoption of learner-centred approaches. Finally, the frequent use of the native language (Amharic) in English lessons, driven by the need to enhance comprehension, inadvertently impeded the development of communicative competence. These findings are consistent with prior research [34,43–46], which underscores the necessity of strengthening curriculum-based strategies to promote effective English language instruction.

The scarcity of teaching resources emerged as a significant barrier. It could limit teachers’ ability to effectively implement innovative teaching strategies. The absence of digital literacy tools and other resources essential for fostering communication skills further constrained the adoption of innovative teaching methodologies. These challenges align with previous research findings [34,42,43,44,45,47]. These studies underscored similar obstacles in the implementation of innovative language teaching strategies in primary education.

## 8. Conclusion

Recent advancements in language pedagogy underline the necessity of criticality and innovations. It promotes the application of innovative teaching strategies that enable learners improving their language skills. EFL teachers have the role of shaping linguistic and cognitive outcomes of students at an early age. They ensure that pedagogical approaches remain adaptive, effective, and aligned with learners’ educational needs.

The classroom implementation of teaching strategies in primary schools, however, revealed notable deficiencies. The findings in this study indicated that primary school EFL teachers face considerable challenges to use innovative teaching strategies. Economic constraints pose significant challenges. However, teachers must explore alternative and cost-effective pedagogical approaches. It was observed that they were over-reliance on uniform teaching strategies across all lessons and content. The prevalent instructional approaches often lack elements that foster critical thinking and problem-solving skills. Consequently, their instruction was frequently limited to cognitive aspects of language learning, with an observable bias toward grammar and vocabulary at the expense of oral skills such as listening and speaking.

Teacher-centred methodologies continue to dominate with students primarily assuming passive roles as listeners rather than active participants in the learning process. This approach restricts student interaction and engagement. Consequently, the teaching strategies employed in primary EFL classrooms were often ineffective. Lesson objectives were not adequately communicated or aligned with students’ language learning needs. To address these problems, innovative teaching strategies such as task-based and project-based learning, multimedia resources (audio, video, and audiovisual aids), digital presentations, differentiated instruction, and interactive activities (e.g., games) must be incorporated into classroom instruction. They foster dynamic and engaging learning environments, and students will be encouraged to use English in meaningful and authentic contexts.

Moreover, teachers’ pedagogical practices were not consistently guided by the curriculum and syllabus. Instructional planning is often confined to textbook reading and routine lesson planning, which resulted a misalignment between teaching strategies and curricular expectations. A well-structured curriculum allows instructional flexibility that fosters innovative teaching strategies. Such a curriculum should encourage the integration of diverse teaching materials and immersive learning experiences through technology, games, and collaborative projects, thereby enhancing both language acquisition and pedagogical creativity.

Teachers have recognized the benefits of innovative teaching strategies though they reported significant challenges in their implementation. Many of them possess limited pedagogical knowledge to effectively incorporate contemporary language teaching techniques. Additionally, a misalignment between textbook content and students’ learning circumstances often results in disengagement and frustration among learners. Contextually relevant language inputs, derived from students’ daily experiences are necessary to make learning more accessible and meaningful. Furthermore, the scarcity of teaching resources aggravates these challenges, limiting opportunities for interactive and communicative instruction.

Hence, it is concluded that primary school EFL teachers did not effectively implement innovative ELT strategies. It led to a disconnection between classroom instruction and curriculum requirements. Despite their recognized benefits, the application of the strategies in Ethiopian primary schools remains limited due to different challenges. The challenges collectively hindered the adoption of interactive and innovative ELT strategies. Therefore, it necessitates a systemic reform to support teacher development and the integration of effective language teaching strategies.

## 9. Recommendation

To teach English effectively, teachers are responsible fitting their teaching strategies with students learning styles and preferences. Hence, innovative ELT strategies should be practised in classrooms though there are hindrances. The challenges to use these strategies should be addressed that the researchers recommend the following suggestions for different stakeholders.

- Engaging teachers in curriculum and training development improves the relevance and effectiveness of English language instruction in Ethiopia.
- The Ministry of Education and Regional Education Bureaus should provide regular training workshops on innovative teaching strategies. Promoting teacher collaboration and knowledge-sharing fosters a sustainable professional learning community.
- Equipping classrooms with diverse teaching materials and technological tools boosts interactive learning.
- ELT policies should be periodically reviewed and adapted to align with innovative teaching strategies in the Ethiopian context.
- Offering incentives for English teachers who adopt and adapt innovative teaching practices can enhance motivation and professional engagement.

## 10. Limitations of the study

The study advances understanding of innovative ELT strategies in primary school EFL classrooms. However, it has challenged with the following limitations:

- The study’s generalization is constrained by its limited sample of teachers from ten schools, potentially limiting its applicability to broader educational contexts. Future research should incorporate a more diverse sample across various geographical settings to enhance representativeness.
- The short-term data collection captures only a snapshot of instructional practices, overlooking their dynamic nature. Longitudinal studies are needed to examine how pedagogical approaches evolve in response to professional development, classroom experiences, and policy changes.
- The study did not assess student learning outcomes, leaving the impact of innovative strategies on engagement, language proficiency, and retention unexplored. Future research should address this gap by examining the direct effects on student performance.
- Institutional constraints-such as standardized testing, curricular mandates, and disparities in professional development and technological access influences teachers’ ability to implement innovative strategies. Further investigation is needed to explore how these external factors shape the adoption of creative teaching strategies in EFL classrooms.

## Supporting information

S1 Appendix. (DOCX)

## References

[1] Boboyeva FF. Modern Approaches to Teaching English: Effective Strategies for Classroom Success. Academic Research in Educational Sciences. 2024;5(11):265–70.

[2] Ismailjanovna MS, Makhmudzjanovna RF, Mamatkodirovich KN, Maxmudjonovna UM, Odiljonovna TB. Integration of cognitive and emotional aspects in the process of teaching foreign languages to primary school children. Library of Progress-Library Science, Information Technology & Computer. 2024;44(3).

[3] Richards JC, Rodgers TS. Approaches and methods in language teaching. Cambridge University Press; 2014.

[4] Kumaravadivelu B. Beyond methods: Macrostrategies for language teaching. Yale University Press; 2003.

[5] Killen R, O’Toole M. Effective teaching strategies 8e. Cengage AU; 2023.

[6] Nepal A. Pedagogical shift in ELT through post-method pedagogy. Research Journal. 2023;8(1):1–1.

[7] John S. Language Teaching: Refashioning the Role of the English Teacher. Journal of the Faculty of Education. 2021;11(17):69–82.

[8] Miller CM, Ndebele DH, Patel SW. The acquisition of language by children: how do children learn language so quickly and effortlessly? Literature and Linguistics Journal. 2023;2(1).

[9] Tiruneh D, Hoddinott J, Rolleston C, Sabates R, Woldehanna T. Understanding achievement in numeracy among primary school children in Ethiopia: evidence from RISE Ethiopia study. RISE Working Paper Series; 2021. p. 21/071. doi: 10.35489/BSG-RISE-WP_2021/071

[10] Molla T, Tiruneh DT. Ethiopia’s education system is in crisis–now’s the time to fix it. The Conversation. 2023.

[11] Barnett C, Copland F, S G. Global practices in teaching English to young learners: Ten years on. 2024.

[12] Paragae IP. Innovative teaching strategies in teaching English as a foreign language. ETLiJ. 2023;4(1):1–9.

[13] Fabregas ID, Zarei N. Innovative and creative English teaching strategies: a conceptual framework. Recoletos Multidisciplinary Research Journal. 2024;12(1):73–84.

[14] Constance B, Pierre R. A phenomenological insight into the use of pandemic pedagogy in the primary English language arts grammar class. 2024.

[15] Daligdig M, Edrozo R, Galang R, Lambayan J, Mara MG, Vacalares ST. Innovative Strategies for Teaching English in the Wake of COVID-19 Pandemic: A Phenomenological Study. CJMS. 2023;3(6):87–95. doi: 10.47760/cognizance.2023.v03i06.006

[16] Kasper M, Mikk J, Uibu K. Language teaching strategies’ impact on third-grade students’ reading outcomes and reading interest. International Electronic Journal of Elementary Education. 2018;10(5):601–10.

[17] Bernaus M, Gardner RC, Wilson A. Teachers’ motivation, classroom strategy use, students’ motivation and second language achievement. 2009.

[18] Isbell R, Lindauer L, Lowrance A, Sobol J. The effects of storytelling and story reading on the oral language complexity and story comprehension of young children. Early Childhood Education Journal. 2004;32:157–63.

[19] Jaelani A, Utami IR. The implementation of Just A Minute (JAM) technique to scaffold students’ speaking fluency: A case study. English Journal. 2020;14(1):1–5.

[20] Adams D, Carlson H, Hamm M. Cooperative learning & educational media: collaborating with technology and each other. Educational Technology. 1990.

[21] Yaccob NS, Yunus MM. Language games in teaching and learning English grammar: A literature review. Arab World English Journal. 2019;10(1):209–17.

[22] Schmidt R. Attention, awareness, and individual differences in language learning. Perspectives on individual characteristics and foreign language education. 2012;6(27):27–49.

[23] Eggins H. Globalization and reform in higher education. McGraw-Hill Education (UK); 2003.

[24] Cheng AY, Cheng MM, Tang SY. Differences in pedagogical understanding among student–teachers in a four-year initial teacher education programme. Teachers and Teaching. 2014;20(2).

[25] Falloon G. What works and what doesn’t with Digital Learning Objects (DLOs): The Microsoft Partners in Learning (NZ)-Project Milo. In International Conference on Educational Technology (3rd: 2006); 2006. p. 32–48.

[26] Guskey TR. Evaluating professional development. Corwin Press; 2000.

[27] Creswell JD, Creswell JW. Research design: Qualitative, quantitative, and mixed methods approaches. Sage Publications; 2017.

[28] Bowen GA. Document analysis as a qualitative research method. Qualitative Research Journal. 2009;9(2):27–40.

[29] Landis JR, Koch GG. An application of hierarchical kappa-type statistics in the assessment of majority agreement among multiple observers. Biometrics. 1977:363–74. 884196

[30] The Education Hub. A brief introduction to direct instruction. 2023. https://www.theeducationhub.org.nz

[31] Scrivener J. The essential guide to English language teaching. [Cited 26.12. 2023]. https://www.ircambridge.com/books/Learning_Teaching.pdf

[32] Copland F, Garton S, López-Gopar M, Makhmudova N, Meke ES, Rahman A. English as a subject in primary school: Lessons from Bangladesh, Malawi, Mexico and Uzbekistan. 2024.

[33] Berlie AD, Ukumo EY. Primary school English teachers’ practice of teaching early grade reading. Social Sciences & Humanities Open. 2024;9:100840.

[34] Clare M, Graham S. Strategies of teaching English as a second language in multilingual classrooms in Meheba refugee settlement in Kalumbila District of Zambia. British Journal of Multidisciplinary and Advanced Studies. 2024;5(1):79–89.

[35] Bwire AM, Mugo TM. Strategies used in teaching English language oral skills and effects on primary school learner participation in Embu County, Kenya. 2024.

[36] Sun Y. Fostering interactive language use: Strategies and challenges in English language teaching. Lecture Notes in Education Psychology and Public Media. 2024;54:10–5.

[37] Singh RB. Navigating English language education challenges in resource-limited contexts. KMC Journal. 2024;6(1):135–52.

[38] Negash T. Education in Ethiopia: From crisis to the brink of collapse. Nordiska Afrikainstitutet. 2006.

[39] MOE. Education Sector Development Programme VI (ESDP VI). Addis Ababa: Ministry of Education. 2021.

[40] Areaya S, Tefera B, Tefera D. A survey of primary school English language teachers’ language competency in Ethiopia. Ethiopian Journal Of Behavioural Studies. 2021;4(2):110–31.

[41] Aweke F. Current issues of English language teaching in Ethiopia: A system loop evaluation of the current English language teaching in Ethiopia. 2019.

[42] Jamalova M. Integrating modern technology in English language teaching: Innovations and outcomes in school education. Eurasian Science Review An International Peer-Reviewed Multidisciplinary Journal. 2024;2(2):138–42.

[43] MOE. General Education Curriculum Framework. Addis Ababa. 2020.

[44] Fitria D. The influence of teachers’ teaching strategy on student motivation in learning English. UIN Ar-Raniry. 2023.

[45] Ingabire MY, Mukingambeho D, Nizeyimana G, Tusiime M. Pedagogical strategies limitations and practices leading to improved teacher-learners’ classroom interactions through English in lower primary schools of Rwanda. African Journal of Empirical Research. 2024;5(2):813–23.

[46] Kumar M. Challenges and solutions in English language teaching (ELT) in rural settings: A case study in India. Research Review International Journal of Multidisciplinary. 2024;9(1):75–82.

[47] Rahmaniar R, Sardi A, Surahmat Z, Nurnaifah II. Challenge and opportunities: A qualitative exploration of junior high school English language educators’ perspectives on implementing differentiated instruction. JELITA. 2024;5(1):28–40.

