# Supplementary material for: Examining the application of innovative ELT strategies in primary school EFL classrooms
Source: PLoS One. 2025 Aug 25;20(8):e0328902. doi: 10.1371/journal.pone.0328902 (PMC12377625; doi:10.1371/journal.pone.0328902)
Supplement: S1 — (DOCX) [file pone.0328902.s001.docx]

Appendix

1. **Questionnaire**
   1. Dear respondents, please write your responses for each question based on its contents.
2. Which teaching strategies do you commonly use in your English classes?

………………………………………………………………………………………………………………………………………………………………………………………………………………

1. Are there any specific teaching strategies that you are particularly motivated to use? Why?

……………………………………………………………………………………………………………………………………………………………………………………………………………………………………………………………………………………………………….

1. How effective do you find these strategies in enhancing student learning? (Rate 1-5)

…………………………………………………………………………………………………………………………………………………………………………………………………………………………………………………………………………………………………………

1. How do you balance your personal teaching preferences with the requirements of the curriculum and school policies?

…………………………………………………………………………………………………………………………………………………………………………………………………………………………………………………………………………………………………………

1. Are there any specific teaching strategies that you find challenging to implement? Why?

…………………………………………………………………………………………………………………………………………………………………………………………………………………………………………………………………………………………………………

1. What challenges do you face when implementing these strategies?

…………………………………………………………………………………………………………………………………………………………………………………………………………………………………………………………………………………………………………

1. What benefits have you observed from using innovative teaching strategies?

…………………………………………………………………………………………………………………………………………………………………………………………………………………………………………………………………………………………………………

1. To what extent do you believe your teaching strategies align with contemporary language teaching approaches? (Rate 1-5)

…………………………………………………………………………………………………………………………………………………………………………………………………………………………………………………………………………………………………………

1. What resources do you utilize to inform your teaching strategy practices?

…………………………………………………………………………………………………………………………………………………………………………………………………………………………………………………………………………………………………………

1. **Classroom observation**

| Observation Criteria | Responses | | Comments |
| --- | --- | --- | --- |
| Lesson Structure and Organization | Yes | No |  |
| The Lesson has a clear and logical flow. |  |  |  |
| The Lesson objectives are communicated to students. |  |  |  |
| The Lesson is well-paced and timed appropriately. |  |  |  |
| Teaching Strategies |  |  |  |
| Direct Instruction |  |  |  |
| Lecture/Explanation |  |  |  |
| Modelling/Demonstration |  |  |  |
| Interactive Instruction |  |  |  |
| Questioning and Discussion |  |  |  |
| Teacher-led Whole Class Discussion |  |  |  |
| Cooperative Learning |  |  |  |
| Pair Work |  |  |  |
| Small Group Activities |  |  |  |
| Peer Tutoring |  |  |  |
| Differentiated Instruction |  |  |  |
| Tiered Activities |  |  |  |
| Learning Stations |  |  |  |
| Personalized Learning |  |  |  |
| Task-Based Learning |  |  |  |
| Problem-Solving Activities |  |  |  |
| Project-Based Learning |  |  |  |
| Simulations and Role-Plays |  |  |  |
| Technology Integration |  |  |  |
| Digital Presentations |  |  |  |
| Educational Apps and Software |  |  |  |
| Online Resources and Tools |  |  |  |
| Multisensory Approaches |  |  |  |
| Visual Aids (e.g., charts, posters) |  |  |  |
| Manipulatives and Tactile Activities |  |  |  |
| Audio/Video Resources |  |  |  |
| Student Engagement |  |  |  |
| Students are actively participating in the lesson. |  |  |  |
| Students demonstrate an understanding of the content. |  |  |  |
| Students are engaged and on-task throughout the lesson. |  |  |  |
| Classroom Management |  |  |  |
| The classroom environment is conducive to learning. |  |  |  |
| The teacher effectively manages student behaviour. |  |  |  |
| Transitions between activities are smooth and efficient. |  |  |  |
| Classroom Environment |  |  |  |
| Is the classroom arranged in a conducive learning environment? |  |  |  |
| Are the walls decorated with English language resources? |  |  |  |
| Are there enough English textbooks and supplementary materials? |  |  |  |
| Is the teacher's English language proficiency evident in the classroom? |  |  |  |

1. **Document analysis**

|  | Document Type | Criteria | Details to Analyze | Notes |
| --- | --- | --- | --- | --- |
|  | Lesson Plans | Goals and Objectives | Alignment with motivational aspects (e.g., engagement, relevance) |  |
|  |  | Instructional Strategies | Types of strategies planned (e.g., group work, games, direct instruction) |  |
|  |  | Assessment Methods | Methods for evaluating student understanding and progress |  |
|  |  | Adaptations | Adjustments for different learner needs and levels |  |
|  | Teaching Materials | Variety and Appropriateness | Diversity of materials used (e.g., worksheets, multimedia resources) |  |
|  |  | Student Engagement | How materials encourage interaction and engagement |  |
|  |  | Motivational Aspects | Materials designed to enhance student motivation (e.g., games, real-life applications) |  |
|  | Classroom Activities | Activity Types | Nature of activities (e.g., cooperative learning, projects, individual tasks) |  |
|  |  | Student-Centered vs. Teacher-Centered | Balance between student-driven and teacher-led activities |  |
|  |  | Motivational Techniques | Activities designed to boost student motivation (e.g., rewards, praise) |  |
|  | Teacher Reflection Journals/Self-Evaluations | Reflections on Motivation | Teacher's perception of their motivation and impact on teaching |  |
|  |  | Evaluation of Strategies | Teacher's assessment of strategy effectiveness and adjustments made |  |
|  |  | Challenges and Successes | Personal reflections on challenges faced and successes achieved. |  |
|  | Professional Development Records | Training Attended | Types of professional development activities and relevance to teaching |  |
|  |  | Application of New Knowledge | Evidence of incorporation of new skills or knowledge into teaching |  |
|  |  | Reflection on Growth | Teacher's reflections on growth and its impact on teaching practices |  |

1. **Coding Framework / Rubric**

| **Theme Category** | **Definition** | **Example Code** |
| --- | --- | --- |
| Application of Innovative Teaching Strategies | Use of new or creative teaching methods in EFL instruction | Group work, storytelling, peer teaching |
| Challenges in Strategy Implementation | Obstacles teachers face while using innovative strategies | Lack of resources, large class size |
| Perceived Benefits of Innovation | Reported outcomes or advantages of using new strategies | Improved student engagement, better comprehension |
| Alignment with Contemporary ELT Approaches | How teaching strategies reflect modern ELT principles (CLT, task-based, etc.) | Student-centered, communicative tasks |
